# Correlation Between Chest CT Severity Scores and Glycosylated Haemoglobin Levels and its Outcome in Patients With COVID-19: A Retrospective Study in a Tertiary Care Hospital

**DOI:** 10.7759/cureus.28371

**Published:** 2022-08-25

**Authors:** Gaurav Sahu, Shiv H Joshi, Sarthak Mendiratta

**Affiliations:** 1 Department of Medicine, Jawaharlal Nehru Medical College, Datta Meghe Institute of Medical Sciences (Deemed to be University), Wardha, IND; 2 Department of Community Medicine, Jawaharlal Nehru Medical College, Datta Meghe Institute of Medical Sciences (Deemed to be University), Wardha, IND

**Keywords:** hrct score, computed tomography, hba1c, diabetes, covid-19

## Abstract

Background

In this study, we aimed to compare the imaging findings between coronavirus disease (COVID-19) patients with well-controlled, poorly-controlled, and non-diabetic patients and subsequently find any relation between haemoglobin A1c (HbA1c) levels and high-resolution chest computed tomography (HRCT) chest score.

Methodology

A total of 200 individuals with coexisting COVID-19 and type 2 diabetes mellitus were included in this retrospective cohort study. Based on their HbA1c levels, patients were divided into three groups. The imaging data and laboratory values were obtained from the online medical records of the patients. In addition, the chest computed tomography (CT) score was evaluated as the sum of individual scores from five lung lobes: scores of 0, 1, 2, 3, 4, and 5 were assigned to each lobe. Any peripheral opacification pattern was noted. Haemoglobin A1c (HbA1c) levels and HRCT scores were then analysed by multiple linear regression models using R software.

Results

The prevalence of diabetes in the study population was 71.5%. Of this, 56 patients had well-controlled diabetes (28%) and 87 patients had poorly controlled diabetes (43.5%); 126 (63%) patients were male and the median age was 54.45 years (95% CI: 54.45 ± 15.53). We found that diabetes status, co-presence of ground-glass appearance with mixed consolidation, and consolidation and reverse halo sign in the HRCT findings were significant predictors of the HRCT scores in patients with COVID-19.

Conclusions

The presence of any co-morbidities should be viewed as a high-risk case of COVID-19. Diabetes status is significantly associated with the severity of HRCT findings in lab-confirmed COVID-19 infection. Therefore, it is important to prioritise the patients who have COVID-19 along with diabetes.

## Introduction

The 2019 novel coronavirus (2019-nCoV), formerly known as the severe acute respiratory syndrome coronavirus 2 (SARS-CoV-2), is a rapidly evolving zoonotic agent that first surfaced in December 2019 in Wuhan, China, and causes the coronavirus disease 2019 (COVID-19) [1]. This virus causes a syndrome that can progress to a critical care pulmonary condition in some patients, necessitating specialised management in intensive care units (ICU) in many hospitals [2].

Coronaviruses are a group of positive-sense single-stranded RNA viruses that infects birds, animals, and humans, causing respiratory, hepatic, neurological, and gastrointestinal problems [3]. SARS-CoV-2 is a distinct member of this family that causes pneumonia, a severe COVID-19 acute respiratory illness. SARS-CoV-2 is made up of numerous proteins, including the nucleocapsid, spike, membrane, and envelope. Most importantly, the spike protein is crucial because it allows the virus to enter and infect host cells and determine viral pathogenicity, host tropism, and illness [4].

The clinical signs and symptoms are similar to those of influenza. They can vary from asymptomatic/paucisymptomatic to severe constitutional symptoms like fever, breathlessness, and cough which are more frequently associated, compared to anorexia, arthralgia, nausea, vomiting, and diarrhoea which are less prevalent. However, the majority of the population is asymptomatic. SARS-CoV-2 can progress to detrimental sequelae, leading to acute respiratory distress syndrome (ARDS), pneumonia, multi-organ failure, and disseminated intravascular coagulation, all of which can lead to death [5].

This COVID-19 pandemic has successfully reflected how ill-prepared our healthcare system of India and the government were in terms of technology, which is outmoded and underfunded, to respond quickly. These challenges compelled industries, private sectors, and healthcare authorities to prioritise the critical need to develop specific tests for early and accurate detection and diagnosis of the disease to manage and stop its spread effectively. The most effective technique for detecting SARS-CoV-2 is real-time reverse transcription-polymerase chain reaction (RT-PCR) [6]. On the other hand, high-resolution chest computed tomography (HRCT) imaging has played an important role in diagnosing and managing suspected or confirmed cases of COVID-19 patients [7].

To determine the severity of the illness early, HRCT chest was advised [8]. Peripherally distributed findings like ground-glass opacities (GGO) and consolidation were commonly observed in the majority of SARS-CoV-2 infected individuals [9,10]. According to previous literature, severe COVID-19 infection prove to have higher HRCT severity scores as compared to mild to moderate forms of the infection [11].

According to some previous research, patients with comorbidities such as diabetes, cardiovascular disease, and high blood pressure, as well as those who are older, had poorer clinical results and a greater risk of COVID-19-related sequelae [12,13].

Diabetes mellitus is a significant contributor to the global morbidity charts, and it has the undoubtedly high potential to disrupt every physiological system of the human body [14]. As a result, a dysregulated immune system may develop, making diabetic people vulnerable to a wide spectrum of other diseases [15].

The role of glycaemic management in individuals having coexistent COVID-19 and diabetes is hazy and unclear in providing prognostic outcomes. Therefore, we aimed to analyse diabetic blood picture status and imaging reports of lab-confirmed COVID-19 patients and intend to establish a relation between the two attributes.

The purpose of this study is to find an association between glycemic control in known diabetes mellitus patients and its imaging outcome in confirmed COVID-19 patients and to find a correlation, if any, between HbA1c levels and HRCT scores.

## Materials and methods

We conducted a retrospective cohort study in a tertiary care central rural hospital in Vidarbha Region, Maharashtra. Vidarbha is located in the northeastern part of the state comprising the Amravati division and Nagpur division collectively formed by 11 districts. Acharya Vinoba Bhave Rural Hospital caters to this region with a population of more than 2 million. A total of 532 COVID-confirmed patients were admitted to the hospital between April 2021 and June 2021, of which 200 patients were included in our study who had a confirmed RT-PCR report for COVID-19 infection, along with their HRCT and HbA1c report investigated within the period of study. Patients who were younger than 18 years or older than 75 years were excluded from the study. To nullify the associations of other comorbid conditions, the study included only those COVID-19-positive patients with no other chronic metabolic diseases other than type 2 diabetes mellitus.

According to the American Diabetes Association's recommended glycosylated HbA1c levels, the patients were grouped into three attributes: non-diabetic, well-controlled (pre-diabetic), and poorly controlled diabetes [16]. Patients having HbA1c > 6.5% were grouped under poorly controlled diabetes, whereas those with HbA1c levels falling between 5.7 and less than 6.5% were classified as having pre-diabetes or diabetes. Those who had Hb1Ac levels less than 5.7% were grouped under non-diabetic as controls of the study. 

Study procedures

Demographics and Laboratory Tests

This Hospital is equipped with an online central information database software that can be accessed by logging into the hospital information system (HIS) domain secured and restricted within the hospital perimeter. Every patient has a unique identification number by which all the information regarding personal information, socio-demographic details, medical history, laboratory findings, radiographic scan reports including HRCT and X-rays, treatment progress, and the outcome were retrieved. The laboratory findings included the basic routine investigation workup along with random blood sugar (RBS) and HbA1c levels. Radiological chest HRCT reports were retrieved and were studied by an expert radiologist to prevent any bias, and visually scored according to the CO-RADS scoring system and any peripheral CT pattern if this was present.

Chest CT Interpretation

All patients were investigated for chest CT scans after admission, which then were reported by the same radiologist (with years of experience in CT imaging) to prevent any bias. Individual scores from each of the five lung lobes were added to calculate the chest CT severity score. Based on the percentage of pulmonary involvement, each lobe was given a score from 0 to 5 [17]. The total severity score varied from 0 to 25 (severe involvement). The five most common patterns seen in chest CT imaging were GGO, consolidation, GGO/consolidation (mixed), reverse halo sign, and crazy-paving.

Data Analysis

Participants’ lab reports were retrieved retrospectively from the central online database-HIS of our hospital, using the patient’s unique identification number and the data was extracted into the Microsoft Excel file format. It was then analysed in R version 4.0.3 (R Foundation for Statistical Computing, Vienna, Austria). Frequency distribution and cross-tabulations were used to summarise the socio-demographic data. Depending on whether the data were parametric or non-parametric, appropriate statistical tests of significance were applied. Further, multivariate analysis was done in the form of linear regression analysis to answer our objectives. To compare the median HRCT scores according to the diabetes status of the study participants we used the Kruskal-Wallis rank sum test. The P-value of less than 0.05 was considered statistically significant for all the tests used.

## Results

Demographic findings

Patient information was collected from a total of 200 participants out of 532 confirmed cases of COVID-19. Of the initial 532 cases enrolled, 332 patients with COVID-19 were excluded from the study, including 58 patients younger than 18 or older than 75 years old, 214 patients without complete medical records, and 60 had other comorbidities, with hypertension having the highest prevalence among them. In the present study, 126 (63%) were males and 74 (37%) were females. The prevalence of diabetes in the study population was 71.5%, of which 56 patients had well-controlled diabetes (28%) and 87 patients had poorly controlled diabetes (43.5%). This study included SARS-CoV-2-infected patients who were 18 to 75 years old, with a mean age of 54.45 (95% CI: 54.45 ± 15.53). The mean age of the uncontrolled diabetic group was 51.33 years (95% CI: 51.33 ± 15.44), whereas the mean age in the controlled diabetic group was 57.30 years (95% CI: 57.30 ± 14.56), and the difference was moderately significant (P = 0.04), as shown in Table 1.

**Table 1:** Socio-demographic variables and diabetic status of the patients. SD: Standard deviation.

Variables	Total	Non-diabetic	Prediabetic	Diabetic	P-value
n (%)	200 (100%)	57 (28.5%)	56 (28.0%)	87 (43.5%)	
Age (years)					
Mean±SD	54.45±15.53 (18-75)	56.42 ±15.98 (18-75)	57.30±14.56 (32-75)	51.33±15.44 (18-75)	0.04
Gender					
Male, n (%)	126 (63%)	38 (33.33%)	37 (66.07%)	51 (58.62%)	0.44
Female, n (%)	74 (37%)	19 (66.67%)	19 (33.93%)	36 (41.38%)	

The distribution of male and female patients was non-significant between the two groups (P = 0.44). Even though the study population was male-dominant, the non-diabetic group had more females (66.67 %). Whereas in the well-controlled (66.07 %) and poorly-controlled (58.62 %) diabetic group, male patients constituted a higher percentage.

Imaging findings

Table 2 summarises the HRCT imaging findings. The median total HRCT severity score was 14 having a cumulative range from 1 to 25. The median score was 6, 14 and 16 in non-diabetic, well-controlled, and poorly controlled diabetes, respectively (P < 0.001). Data regarding the scores of each lobe was also retrieved and compared with the three main domains. The total median score for the right upper lobe was 3, for the right middle lobe was 3, for the right lower lobe was 3, for the left upper lobe was 3 and for the left lower lobe was 2. In addition to this, the predominant CT pattern was also analysed. The most common CT finding was the GGO pattern (78.50%), followed by GGO/consolidation mixed (37%), consolidation alone (22.50%), crazy pavement (7.00%) and reverse halo pattern (2.00%). These findings were significantly associated with the diabetic status and the HbA1C level of the individuals and had a positive correlation.

**Table 2:** Comparison between high resolution computed tomography (HRCT) findings and clinical diabetic status of the patients. HRCT: high-resolution computed tomography; RUL: right upper lobe; RML: right middle lobe; RLL: right lower lobe; LUL: left upper lobe; LLL: left lower lobe; GGO: ground-glass opacification.

Variables	Total	Non-diabetic	Prediabetic	Diabetic	P-value
n (%)	200 (100%)	57 (28.5%)	56 (28.0%)	87 (43.5%)	
Median total HRCT Score	14 (1-25)	6 (2-22)	14 (8-25)	16 (1-25)	<0.001*
HRCT score according to lobe					
RUL median	3 (0-5)	1±0.86 (0-4)	3±1.05 (0-5)	3±0.91 (2-5)	<0.0001*
RML median	3 (0-5)	1±0.91 (0-4)	3±1.09 (0-5)	3±0.95 (1-5)	<0.0001*
RLL median	3 (0-5)	1±1.38 (0-5)	4±1.26 (0-5)	4±1.18 (0-5)	<0.0001*
LUL median	3 (0-5)	1±1.23 (0-5)	3±1.47 (0-5)	3±1.23 (0-5)	<0.0001*
LLL median	2 (0-5)	1±1.09 (0-5)	2±1.54 (0-5)	3±1.28 (0-5)	<0.0001*
Predominant CT pattern					
GGO, n (%)	157 (78.50%)	50 (87.72%)	43 (76.79%)	64 (73.56%)	<0.001*
Consolidation, n (%)	45 (22.50%)	1 (1.75%)	9 (16.07%)	35 (40.23%)	
GGO/Consolidation (mixed), n (%)	74 (37.00%)	4 (7.02%)	19 (33.93%)	51 (58.62%)	<0.001*
Crazy pavement, n (%)	14 (7.00%)	0 (0.00%)	2 (3.57%)	12 (13.79%)	
Reverse halo sign, n (%)	4 (2.00%)	0 (0.00%)	2 (3.57%)	2 (2.30%)	

We used multiple linear regression analysis to determine predictors of HRCT score. All variables were entered in R software to choose a model by the generalized Akaike Information Criterion (AIC) in a stepwise method. The direction in the step function was set to “both.” The estimate was obtained for each of the variables in the fitted model where a p-value of 0.05 was considered significant. Two models were made which were completely identical, except that the first model included the diabetes status as a categorical variable, whereas the second model included HbA1c values. The final output from model 1 had the lowest AIC value of 572.25, and a residual standard error of 4.13 on 195 degrees of freedom. The Nagelkerke Pseudo R-squared value of our final model is 0.5226 indicating an explainable variation of 52.26%. We found that diabetes status, co-presence of GGO with consolidation, and reverse halo sign in the HRCT findings were significant predictors of the HRCT scores in patients with COVID-19.

The final output from model 2 had the lowest AIC value of 572.25 and a residual standard error of 4.793 on 195 degrees of freedom. The Nagelkerke Pseudo R-squared value of our final model is 0.3569 indicating an explainable variation of 35.69%. We found that HbA1c levels, co-presence of GGO with consolidation, consolidation, and reverse halo sign in the HRCT findings were significant predictors of the HRCT scores in patients with COVID-19 (Tables 3, 4).

**Table 3:** Multiple linear regression analysis model 1. GGO: ground-glass opacification. Significant codes: 0 ‘***’ 0.001 ‘**’ 0.01 ‘*’ 0.05 ‘.’ 0.1 ‘ ’ 1 Residual standard error: 4.13 on 195 degrees of freedom Multiple R-squared: 0.5226,  Adjusted R-squared: 0.5128 F-statistic: 53.36 on 4 and 195 degrees of freedom, P-value: < 2.2e-16

Model 1					
Coefficients:	Estimate Std	Error	t value	Pr(>|t|)	
(Intercept)	14.1680	0.5945	23.833	< 2e-16	***
Diabetic-non-diabetic	-7.4021	0.7855	-9.423	< 2e-16	***
Diabetic-pre-diabetic	-0.5368	0.7292	-0.736	0.4625	
GGO and consolidation mixed	3.0853	0.6866	4.494	1.2e-05	***
Reverse halo sign	5.5151	2.1302	2.589	0.0104	*

**Table 4:** Multiple linear regression analysis model 2. GGO: ground-glass opacification. Significant codes: 0 ‘***’ 0.001 ‘**’ 0.01 ‘*’ 0.05 ‘.’ 0.1 ‘ ’ 1 Residual standard error: 4. 793 on 195 degrees of freedom Multiple R-squared: 0.3569, Adjusted R-squared: 0.3437 F-statistic: 27.05 on 4 and 195 degrees of freedom,  P-value < 2.2e-16

Model 2					
Coefficients:	Estimate Std	Error	t value	Pr(>|t|)	
(Intercept)	6.5329	1.1589	5.637	6.00e-08	***
HbA1c levels	0.6346	0.1780	3.565	0.000458	***
GGO and consolidation mixed	4.3966	0.7784	5.648	5.68e-08	***
Consolidation	2.2557	0.8503	2.653	0.008642	**
Reverse Halo	6.8040	2.4761	2.748	0.006561	**

## Discussion

India became the epicentre of the coronavirus pandemic during the period between April and July 2021. During this period, India witnessed a total of 180 million confirmed cases. Out of this, 20% of the total Maharashtra cases were from the Vidarbha region, Nagpur being the major contributor to the statistics. This retrospective cohort study was carried out in a tertiary care hospital located in central rural India that caters to most of the Vidarbha region.

Diabetes is linked to higher mortality and morbidity from various infectious diseases. Some recent studies have found this true for COVID-19 [14,18,19]. Diabetes causes a deterioration in the activation of the Th1 adaptive immune response, which causes a delayed hyperinflammatory response. Studies have seen low levels of CD4 and CD8 and higher levels of Th17, causing an increase in proinflammatory cytokines [20]. The laboratory data and imaging findings of patients with coexisting diabetes who were admitted to our hospital between April and July 2021 were evaluated in this study. We found that higher chest HRCT scores were significantly correlated with higher levels of HbA1C. This can be well understood as diabetes is a significant risk factor in causing the severe spread of the disease in the body as evidenced through imaging findings. A similar result was obtained by Weina Guo and colleagues, they reported that patients with diabetes had considerably higher chest CT scores than those without diabetes [21].

Diabetes has previously been linked to an increased risk of death in COVID-19 patients [13,22]. The significance of hyperglycaemia in the pathogenesis of viral respiratory infections has been noted in several previous studies. Higher blood glucose levels can have a severe impact on pulmonary function, as well as inhibits the immune system and elevates the production of inflammatory cytokines [23,24]. Furthermore, the pancreas expresses angiotensin-converting enzyme 2 (ACE2), one of SARS primary CoV-2 receptors, implying that this novel coronavirus can directly harm pancreatic islets [25]. Nonetheless, more research into the effect of hyperglycaemia on COVID-19 progression is needed. This could explain why COVID-19 patients with diabetes mellitus have an increased risk of severe acute lung damage and ARDS [26]. When RT-PCR testing is not available, in case of delayed test results, or when there is a clinical suspicion of COVID-19 despite initial negative RT-PCR testing, the WHO recommends using radiographic chest imaging as a key tool to establish a diagnostic workup for COVID-19 disease. Clinicians and radiologists should collaborate to create the best imaging modality decision possible [27].

Previous research has shown that CT scores in severe COVID-19 patients were considerably higher than those in mild cases [8,28]. Very few studies have compared this with HbA1C levels. The clinical outcomes and imaging results of 117 lab-confirmed COVID-19 individuals with well-controlled and poorly-controlled diabetes were compared by Raoufi et al. They did not find any substantial difference in chest CT severity levels (P = 0.33) among the well-controlled and poorly-controlled diabetes patients. Furthermore, both groups had similar death and recovery rates (P = 0.54 and P = 0.85, respectively) [29]. Furthermore, GGO was the most common chest CT finding in their research, followed by consolidation (P = 0.08). Our study, on the other hand, showed co-presence of higher HbA1c levels, GGO with consolidation, and reverse halo sign in the HRCT findings to be significant and independent predictors of the HRCT scores in patients of COVID-19. It is also unclear whether HbA1c or Fasting plasma glucose (FPG) levels play a greater role in evaluating the prognosis of COVID-19 patients. Acute viral respiratory infections are linked to a reduction in insulin sensitivity [30]. As a result of which, both well-controlled and poorly-controlled diabetes mellitus show insignificant improvement in clinical outcomes because their FPG levels are raised even after having HbA1c values less than 7%.

The COVID-19 pandemic is overlapping with the pre-existing diabetes pandemic, resulting in large and susceptible populations of COVID-19 and diabetes patients. As a result, additional attention must be paid to these instances during the pandemic to prevent adding to the load on a country's healthcare system.

Limitations

Since this was a retrospective study and involved analysis of the data, we did not assess patients’ blood glucose levels during the course of illness and so, our study is solely based on HbA1c levels. Furthermore, this was a single-centre study, and thus it lacked the external validity required to support the findings and generalise the same in a widespread population. 

## Conclusions

Diabetes is linked to higher mortality and morbidity from various infectious diseases and has been linked to an increased risk of death in COVID-19 patients. Diabetes status is significantly associated with the severity of HRCT findings in lab-confirmed COVID-19 infection. As the severity of HRCT findings has been known to predict the outcome of COVID-affected individuals, it is important to prioritise the patients who have COVID-19 along with diabetes. Moreover, the presence of any co-morbidity should be viewed as a high-risk case of COVID-19. More studies are required to evaluate any co-relation of radiographic scans of confirmed COVID-19 patients and glycated haemoglobin levels with the prognosis of the patient to assess and verify the findings of this study.
